# Knowledge, acceptance and concerns regarding COVID-19 vaccination among pregnant women on the east coast of Peninsular Malaysia: A cross-sectional study

**DOI:** 10.51866/oa.533

**Published:** 2024-06-18

**Authors:** Abu Hanifah Farhana, Nik Rafiza Afendi, Noor Adibah Hanum Che Hashim, Ahmad Amir Ismail, Erinna Mohamad Zon, Abd Rahim Rahimah

**Affiliations:** 1 MBBCh BAO, MMed (O&G), Department of Obstetrics & Gynaecology, School of Medical Sciences, Universiti Sains Malaysia, Kota Bharu, Kelantan, Malaysia. Email: nikrafiza@usm.my; 2 MBBS, Department of Obstetrics & Gynaecology, School of Medical Sciences, Universiti Sains Malaysia, Kota Bharu, Kelantan, Malaysia.; 3 MD, MMed (O&G), Department of Obstetrics & Gynaecology, School of Medical Sciences, Universiti Sains Malaysia, Kota Bharu, Kelantan, Malaysia.; 4 MBBS, MMed (O&G), Department of Obstetrics & Gynaecology, School of Medical Sciences, Universiti Sains Malaysia, Kota Bharu, Kelantan, Malaysia.; 5 MD, Mmed (O&G), Department of Obstetrics & Gynaecology, School of Medical Sciences, Universiti Sains Malaysia, Kota Bharu, Kelantan, Malaysia.; 6 MD, MMed (O&G), Department of Obstetrics & Gynaecology, School of Medical Sciences, Universiti Sains Malaysia, Kota Bharu, Kelantan, Malaysia.

**Keywords:** COVID-19, Pregnant women, Vaccination acceptance, Knowledge, vaccination

## Abstract

**Introduction:**

This prospective cross-sectional study, conducted from 1 April 2022 to 31 October 2022, aimed to assess the knowledge, acceptance and concerns regarding COVID-19 vaccination among pregnant women visiting the Obstetrics and Gynaecology Department of Hospital Universiti Sains Malaysia.

**Methods:**

The study included all pregnant women aged >18 years. Sociodemographic data, information related to COVID-19 and vaccination and information on the knowledge, acceptance and concerns regarding COVID-19 vaccination were collected using a validated questionnaire.

**Results:**

Out of 420 eligible pregnant women, 412 participated in the study, yielding a response rate of 98.1%. Of the respondents, 97.1% had received a COVID-19 vaccine, while 2.9% had not. Approximately 85.2% demonstrated a good understanding of COVID-19 vaccination. Among those vaccinated, 76.8% based their decision on recommendations from healthcare providers or the Ministry of Health. Among those unvaccinated, 91.7% believed that COVID-19 vaccines could harm their pregnancy and baby. Although 51% of the respondents expressed concerns about vaccine safety, 202 still chose to be vaccinated, indicating a willingness to prioritise their health despite apprehensions.

**Conclusion:**

The study found no significant link between acceptance and good knowledge of COVID-19 vaccination. However, income and prior COVID-19 booster vaccination were strongly associated with acceptance. Despite safety concerns, 97.1% of the respondents had received a COVID-19 vaccine. This emphasises the importance of providing comprehensive information and addressing concerns to support informed decision-making among pregnant women. Healthcare providers play a vital role in guiding them through this crucial decision-making process.

## Introduction

COVID-19, originating from a novel coronavirus termed severe acute respiratory syndrome coronavirus 2 (SARS-CoV-2), began in December 2019, initially linked to a Wuhan seafood market.^1^ The World Health Organization (WHO) declared COVID-19 a pandemic on 11 March 2020 due to escalating global cases.^2^ This has left significant marks on societies.

The consequences of COVID-19 are seen in multiple aspects: psychological, social and economic. Pregnant women are at a higher risk for severe illness in the presence of COVID-19. Accumulating evidence indicates that pregnant women are more likely to develop COVID-19-related complications, including the need for invasive ventilation, admission to an intensive care unit and death, than nonpregnant women.^3-5^ However, pregnant women have not been included in any trials on COVID-19 vaccination.

In April 2021, the WHO Strategic Advisory Group of Experts on Immunization recommended that pregnant women can receive a COVID-19 vaccine if the benefits of vaccination outweigh the potential risks, such as occupational activities with an unavoidable high risk of exposure and the presence of comorbidities that place them at a high risk for severe COVID-19.^6^ The American College of Obstetricians and Gynecologists (ACOG) highlighted that vaccination for pregnant women should be considered on a case-by-case basis after consultation between them and their healthcare providers.^7^ Pregnant women should be provided with information about the risks of COVID-19 in pregnancy, the potential benefits of vaccination in the local epidemiological context and the current limitations of vaccine safety data in pregnant women to help them decide. The Ministry of Health (MOH) Malaysia guidelines recommend mRNA vaccines as the preferred option among pregnant and breastfeeding mothers in Malaysia; at the time, Comirnaty by Pfizer was the preferred available vaccine.^8^

At the time of this study, there is no available research on vaccine acceptance among pregnant women locally. In 2020, Azlan et al. investigated public knowledge, attitudes and practices towards COVID-19 in the general Malaysian population rather than in obstetric populations.^9^ Thus, this study aimed to evaluate the knowledge level, acceptance rate and concerns of pregnant women regarding COVID-19 vaccination utilising a validated questionnaire. The findings can be valuable in guiding policymakers in effectively addressing the concerns of pregnant women and educating them about COVID-19 vaccines. Providing accurate information and addressing such concerns can facilitate efforts to improve vaccine acceptance.

## Methods

The sample size for this study was calculated using the single-proportion formula in the Sample Size Calculator (web) by Arifin, with a confidence level of 0.95 and a precision level of 0.05.^10^ The proportion used in this calculation was 30.3% of COVID-19 vaccine acceptance among pregnant mothers.^11^ The sample size calculated considering a 20% dropout rate was 407. Herein, we deliberately collected more samples.

This cross-sectional study was conducted from 1 April 2022 to 31 October 2022. The study employed purposive sampling, targeting pregnant women attending the Obstetrics and Gynaecology Department of Hospital Universiti Sains Malaysia (HUSM). Individuals who met the specified inclusion criteria were approached, and all eligible individuals who expressed willingness to participate were included in the study until the predetermined sample size was achieved. A questionnaire was distributed during the first encounter with pregnant women at the Obstetrics and Gynaecology Department of HUSM.

A total of 420 questionnaires were distributed, and 412 pregnant women completed the questionnaires, yielding a response rate of 98.1%. Pregnant women who were aged >18 years were included, while those who could not understand Malay or English were excluded. The questionnaire consisted of two parts. Part 1 evaluated respondents’ sociodemographic data and information related to COVID-19 and vaccination such as COVID-19 vaccination status (history of receiving a COVID-19 vaccine and booster dose), history of COVID-19, history of COVID-19 among family members and history of receiving previous vaccines other than COVID-19 vaccines. Part 2 consisted of three domains: knowledge, acceptance and concerns regarding COVID-19 vaccination. Additionally, data on the sources from which respondents obtained information about COVID-19 vaccination as well as hesitancy and refusal factors among unvaccinated respondents were collected.

The surveys were conducted using our newly designed validated questionnaire consisting of 24 questions. Eight questions were under the knowledge domain, six items under the acceptance domain and 10 items under the concerns domain, with Cronbach’s alpha values for all factors being more than 0.7 (0.738 for knowledge of benefits, 0.866 for knowledge of side effects and 0.926 for both acceptance and concerns).

For the knowledge domain, the response options were ‘no’, ‘yes’ and ‘do not know’. The scores for correct and incorrect answers were 1 and 0, respectively. Not answering or not knowing was considered neutral and was scored as 0. The maximum possible score was 8. Respondents who obtained scores above the mean total score (≥4.0) were categorised as having good knowledge and those who did not as having poor knowledge.

For the acceptance domain, the questions were scored using a Likert scale as follows: ‘strongly disagree’=1, ‘disagree’=2, ‘neutral’=3, ‘agree’=4 and ‘strongly disagree’=5. The final scores were interpreted as follows: good acceptance (acceptance score equal to or more than the median score) and poor acceptance of COVID-19 vaccination (acceptance score less than the median score).

For the concerns domain, the questions were scored using a Likert scale as follows: ‘not concerned at all’=1, ‘not concerned’=2, ‘a bit concerned’=3, ‘concerned’=4 and ‘very concerned’=5. ‘Very concerned’, ‘concerned’ and ‘a bit concerned’ were combined into one category as ‘concerned’, while ‘not concerned’ and ‘not concerned at all’ were combined into one category as ‘not concerned’.

Data were recorded and analysed using IBM SPSS Statistics (Version 27) Armonk,NY,USA. The sociodemographic data as well as information related to COVID-19 and vaccination of all respondents were analysed using descriptive statistics. Simple and multiple logistic regression analyses were conducted to determine the factors associated with acceptance of COVID-19 vaccination. All variables with P-values of <0.25 in the simple logistic regression analysis were included in the multivariable analysis based on the Wald test by prioritising the important variables to be further evaluated. The level of significance was set at 0.05. The results were presented with crude odds ratios (ORs), adjusted ORs, 95% confidence intervals (CIs) and P-values. The dependent variable was COVID-19 vaccine acceptance, while the independent variables were knowledge, age, BMI, race, occupation, parity, gestation, presence of comorbidities, educational level, income, employment as a healthcare worker, history of COVID-19, history of receiving previous vaccines other than COVID-19 vaccines, history of receiving a COVID-19 vaccine and history of receiving a COVID-19 booster dose.

## Results

### Sociodemographic characteristics and information related to COVID-19 and vaccination

A total of 412 pregnant women answered the questionnaires. The overall mean age and BMI of the respondents were 31.06±5.43 years and 29.24±6.32 kg/m^2^, respectively. Approximately 69.95% of the respondents were in their third trimester of pregnancy. Four hundred respondents (97.1%) had received a COVID-19 vaccine, and 66 (16.0%) had completed the first booster dose (Table 1).

**Table 1:** Sociodemographic characteristics and information related to COVID-19 and vaccination among pregnant women attending the Obstetrics and Gynaecology Department of Hospital Universiti Sains Malaysia (N=412).

Variable	Total N=412 (100%)	Vaccinated n=400 (97.1%)	Unvaccinated n=12 (2.9%)
**Sociodemographic characteristics**			
**Age, year**	31.06 (5.43)^a^	31.1 (5.45)^a^	29.8 (4.99)^a^
**BMI**	29.24 (6.32)^a^	29.27 (6.34)^a^	28.10 (5.97)^a^
**Race**			
Malay	402 (97.6)	390 (97.5)	12 (100.0)
Chinese	6 (1.5)	6 (1.5)	0
Indian	1 (0.2)	1 (0.3)	0
Others	3 (0.7)	3 (0.8)	0
**Occupation**			
Employed	178 (43.2)	174 (43.5)	4 (33.3)
Housewife	225 (54.6)	217 (54.3)	8 (66.7)
Student	9 (2.2)	9 (2.3)	0
**Parity**			
Nulliparous	117 (28.4)	115 (28.7)	2 (16.7)
Multiparous	295 (71.6)	285 (71.3)	10 (83.3)
**Gestation**			
First trimester	13 (3.2)	13 (3.3)	0
Second trimester	111 (26.9)	107 (26.8)	4 (33.3)
Third trimester	288 (69.9)	280 (70.0)	8 (66.7)
**Presence of comorbidities**			
Yes	156 (37.9)	152 (38.0)	4 (33.3)
No	256 (62.1)	248 (62.0)	8 (66.7)
**Educational level**			
Primary	7 (1.7)	6 (1.5)	1 (8.3)
Secondary	158 (38.3)	153 (38.3)	5 (41.7)
Pra-university	136 (33.0)	132 (33.3)	4 (33.3)
Tertiary	111 (26.9)	109 (27.3)	2 (16.7)
**Income** B40 (<RM 4850/month) M40 (RM 4851-10,960/month) T20 (>RM 10,960/month)	335 (81.3) 74 (18.0) 3 (0.7)	324 (81.0) 73 (18.3) 3 (0.8)	11 (91.7) 1 (8.3) 0
**Employment as a healthcare worker** Yes No	50 (12.1) 362 (87.9)	50 (12.5) 350 (87.5)	0 12 (100.0)
**Information related to COVID-19 and vaccination**			
**History of COVID-19** Yes No	124 (30.1) 288 (69.9)	121 (30.3) 279 (69.8)	3 (25.0) 9 (75.0)
**History of COVID-19 among family members** Yes No	155 (37.6) 257 (62.4)	150 (37.5) 250 (62.5)	5 (41.7) 7 (58.3)
**History of receiving previous vaccines other than COVID-19 vaccines** Yes No	237 (57.5) 175 (42.5)	230 (57.5) 170 (42.5)	7 (58.3) 5 (41.7)
**History of receiving COVID-19 booster doses** Yes No	66 (16.0) 346 (84.0)	66 (16.5) 334 (83.5)	0 12 (100.0)

a Mean (standard deviation)

### Knowledge of COVID-19 vaccination

A total of 351 pregnant women (85.2%) had good knowledge of COVID-19 vaccination. Among them, 342 (97.4%) were vaccinated. Two hundred eighty-seven pregnant women (69.7%) answered correctly about the effectiveness of COVID-19 vaccines in reducing COVID-19; 279 of them (97.2%) were vaccinated, while only eight (2.8%) were not. Approximately 68.4% knew that vaccination in pregnant women builds antibodies that might protect the baby, and 81.3% knew that COVID-19 vaccines effectively reduce the severity of infection. More than 60% of the respondents knew all side effects of COVID-19 vaccines. Nine out of the twelve women who were not vaccinated had good knowledge.

### Acceptance of COVID-19 vaccination

Table 2 demonstrates the respondents’ acceptance of COVID-19 vaccination. Of the 412 respondents, 233 exhibited good acceptance of COVID-19 vaccination (56.6%). Among those who had been vaccinated, 228 displayed good acceptance (57%). Conversely, five unvaccinated respondents (41.7%) showed good acceptance.

**Table 2:** Acceptance of COVID-19 vaccination among pregnant women attending the Obstetrics and Gynaecology Department of Hospital Universiti Sains Malaysia (N=412).

Item	Strongly disagree n (%)	Disagree n (%)	Neutral n (%)	Agree n (%)	Strongly agree n (%)
I believe a COVID-19 vaccine is effective in preventing the infection.	20 (4.9)	23 (5.6)	87 (21.1)	181 (43.9)	101 (24.5)
I believe a COVID-19 vaccine will not harm me.	21 (5.1)	29 (7.0)	103 (25.0)	180 (43.7)	79 (19.2)
If my employer or a higher authority mandates vaccination on me, I will receive a COVID-19 vaccine.	22 (5.3)	32 (7.8)	59 (14.3)	210 (51.0)	89 (21.6)
I voluntarily received/will receive a COVID-19 vaccine.	23 (5.6)	23 (5.6)	74 (18.0)	189 (45.9)	103 (25.0)
I will receive/received a COVID-19 vaccine on the recommendation of my family and friends.	25 (6.1)	62 (15.0)	87 (21.1)	177 (43.0)	61 (14.8)
I will receive/received a COVID-19 vaccine on the recommendation from a doctor/the Ministry of Health Malaysia.	21 (5.1)	17 (4.1)	60 (14.6)	224 (54.4)	90 (21.8)

### Concerns about COVID-19 vaccination

Two hundred ten respondents (51.0%) were concerned that COVID-19 vaccination would harm their pregnancy. Despite this, 202 of them were vaccinated. Most of the concerns were regarding vaccine safety, in which 48.1% of the respondents were concerned that vaccination would cause a miscarriage, preterm birth or stillbirth, and 48.5% were concerned that it would cause congenital abnormalities. One hundred ninety-five respondents (47.3%) were concerned about the content or ingredients of a COVID-19 vaccine; however, 187 of them received the vaccination. Of those not vaccinated, 66.7% were mainly concerned about the effect of a COVID-19 vaccine on their pregnancy and baby, the content of such vaccine and the side effects post-vaccination (Table 3).

**Table 3:** Concerns regarding COVID-19 vaccination among pregnant women attending the Obstetrics and Gynaecology Department of Hospital Universiti Sains Malaysia.

	Total (N=412)		Vaccinated (n=400)		Unvaccinated (n=12)	
**Item**	**Concerned n (%)**	**Not concerned n (%)**	**Concerned n (%)**	**Not concerned n (%)**	**Concerned n (%)**	**Not concerned n (%)**
I am concerned that a COVID-19 vaccine would harm my pregnancy and my baby.	210 (51.0)	202 (49.0)	202 (50.5)	198 (49.5)	8 (66.7)	4(33.3)
I am concerned that I will have a miscarriage/preterm birth/stillbirth after I receive a COVID-19 vaccine.	198 (48.1)	214 (51.9)	192 (48.0)	208 (52.0)	6 (50.0)	6 (50.0)
I am worried that a COVID-19 vaccine would cause congenital abnormalities.	200 (48.5)	212 (51.5)	193 (48.3)	207 (51.7)	7 (58.3)	5 (41.7)
I am concerned that a COVID-19 vaccine is dangerous for pregnant women like me.	209 (50.7)	203 (49.3)	200 (50.0)	200 (50.0)	9 (75.0)	3 (25.0)
I am concerned about the contents/ingredients of a COVID-19 vaccine.	195 (47.3)	217 (52.7)	187 (46.8)	213 (53.3)	8 (66.7)	4 (33.3)
I am concerned about the effectiveness of a COVID-19 vaccine.	154 (37.4)	258 (62.6)	149 (37.3)	251 (62.7)	5 (41.7)	7 (58.3)
I am concerned that a COVID-19 vaccine will make me sick or infect me with the COVID-19 virus.	157 (38.1)	255 (61.9)	152 (38.0)	248 (62.0)	5 (41.7)	7 (58.3)
I am concerned that I could spread COVID-19 after I receive a COVID-19 vaccine.	112 (27.2)	300 (72.8)	108 (27.0)	292 (73.0)	4 (33.3)	8 (66.7)
I am concerned about the side effects I may experience after vaccination (e.g. pain, swelling, body ache, fever or allergic reaction).	180 (43.7)	232 (56.3)	172 (43.0)	228 (57.0)	8 (66.7)	4 (33.3)
I am concerned about the information that is being spread about the side effects of a COVID-19 vaccine.	174 (42.2)	238 (57.8)	168 (42.0)	232 (58.0)	6 (50.0)	6 (50.0)

### Factors associated with hesitancy or refusal of COVID-19 vaccination

The most common reasons for hesitancy or refusal of COVID-19 vaccination among the unvaccinated respondents were the belief that vaccination would harm the pregnancy and baby and the lack of information regarding the safety of COVID-19 vaccination for pregnant women (both 91.7%). Approximately 25% of the respondents mentioned that they were not vaccinated because their family members were worried about the effectiveness of a COVID-19 vaccine. None believed that their religion forbids them from receiving COVID-19 vaccination.

### Factors associated with acceptance of COVID-19 vaccination

Table 4 presents the results of the simple logistic regression analysis of the factors associated with acceptance of COVID-19 vaccination. The variables with a P-value of <0.25 were gestation, income, employment as a healthcare worker, history of receiving a previous vaccine other than a COVID-19 vaccine, history of receiving a COVID-19 booster dose and total knowledge score. Table 5 summarises the factors associated with acceptance of COVID-19 vaccination when adjusted for other variables using multiple logistic regression. Two factors – income and history of receiving a COVID-19 booster dose – were significantly associated with acceptance of COVID-19 vaccination. The respondents in the M40 and T20 income groups had 1.88 times higher odds of accepting COVID-19 vaccination (95% CI=1.05, 3.37; P=0.034) when adjusted for other variables. The respondents who had received a COVID-19 booster dose had 1.95 times higher odds of accepting COVID-19 vaccination (95% CI=1.04, 3.63; P=0.036) when adjusted for other variables.

**Table 4:** Simple logistic regression analysis of the factors associated with acceptance of COVID-19 vaccination among pregnant women attending the Obstetrics and Gynaecology Department of Hospital Universiti Sains Malaysia.

Variable	Crude OR (95% CI)	P
**Age, year**		
<25	1	
26-34	1.51 (0.87, 2.64)	0.147
≥ 35	1.24 (o.67, 2.30)	0.499
**BMI, kg/m^2^**	0.99 (0.97, 1.03)	0.772
**Occupation** Employed Housewife	1 0.81 (0.54, 1.19)	0.812
Student	0.85 (0.22, 3.27)	
**Parity** Nulliparous Multiparous	1 1.11 (0.72, 1.71)	0.633
**Gestation**		
First trimester	1	
Second trimester	0.44 (0.12, 1.69)	0.231^*^
Third trimester	0.36 (0.09, 1.33)	0.126^*^
**Presence of comorbidities**		
Yes	1	
No	0.82 (0.55, 1.22)	0.328
**Employment as a healthcare worker**		
No	1	
Yes	1.57 (0.85, 2.93)	0.153^*^
**Income**		
B40	1	
M40 and T20	2.21 (1.29, 3.78)	0.004^*^
**Knowledge score**		
Poor	1	
Good	1.42 (0.82, 2.44)	0.210^*^
**History of COVID-19**		
No	1	
Yes	0.99 (0.65, 1.52)	0.978
**History of COVID-19 among family members**		
No	1	
Yes	1.10 (0.74, 1.65)	0.631
**History of receiving a previous vaccine other than a COVID-19 vaccine**		
No	1	
Yes	1.44 (0.97, 2.13)	0.072^*^
**History of receiving a COVID-19 vaccine**		
No	1	
Yes	1.86 (0.58, 5.95)	0.298
**History of receiving a COVID-19 booster dose** No	1	
Yes	2.32 (1.29, 4.15)	0.005^*^

* P<0.25; OR, odds ratio; CI, confidence interval

**Table 5:** Multiple logistic regression analysis of the factors associated with acceptance of COVID-19 vaccination among pregnant women attending the Obstetrics and Gynaecology Department of Hospital Universiti Sains Malaysia.

Variable	Adjusted OR (95% CI)	P
**Gestation**		
First trimester	1	
Second trimester	0.60 (0.15, 2.42)	0.477
Third trimester	0.52 (0.14, 2.01)	0.373
**Income**		
B40	1	
M40 and T20	1.88 (1.05, 3.37)	0.034^*^
**Employment as a healthcare worker**		
No	1	
Yes	0.83 (0.40, 1.72)	0.616
**History of receiving a previous vaccine other than a COVID-19 vaccine**		
No	1	
Yes	1.24 (0.81, 1.90)	0.325
**History of receiving a COVID-19 booster dose**		
No	1	
Yes	1.95 (1.04, 3.63)	0.036^*^
**Knowledge score**		
Poor	1	
Good	1.24 (0.71, 2.18)	0.447

* P<0.05; OR, odds ratio; CI, confidence interval

## Discussion

Malaysia has provided free COVID-19 vaccination since it became available in the country on 24 February 2021. On 19 May 2023, the MOH Malaysia reported that 86.2% of the Malaysian population had received the first dose of vaccination; 84.4% completed the second dose; only 50.0% received the first booster dose; and only 2.5% received the second booster dose.^12^ However, the exact figures for pregnant women receiving a COVID-19 vaccine are not yet available in any articles or publications. While safety concerns exist for COVID-19 vaccines due to their new technology and the initial exclusion of pregnant women from efficacy trials, emerging data offer reassuring insights into their safety.^13,14^

At the time of the study, the National COVID-19 Immunisation Programme of Malaysia was already in Phase 5, in which the focus was on adolescents aged 12–17 years. Pregnant women were already offered vaccination since Phase 2 (April-August 2021), in which they were categorised to be at a high risk for COVID-19. Among 308 respondents, 74.8% relied on the MOH Malaysia website, 65.0% on social media, and 56.6% on electronic mass media for COVID-19 vaccine information. Social media and electronic mass media emerged as primary information sources, emphasizing their importance in communicating vaccination benefits. Printed media, utilized by only 29.4% of respondents, appeared less effective.

Even though only 56.6% of the pregnant women in this study showed good acceptance of COVID-19 vaccination, 97.1% had completed two doses of COVID-19 vaccination, while 16.0% had received their initial booster dose. These rates align closely with Kalok et al.'s findings who reported a completion rate of 97.8% for both doses, despite our study's majority (81.3%) coming from a low socioeconomic status.^15^ Notably, there's no significant difference in vaccination rates between our demographic and the urban, higher socioeconomic status group studied by Kalok et al. This suggests a high level of COVID-19 vaccination completion across socioeconomic backgrounds, highlighting the effectiveness of vaccination efforts. The figure is also comparable to that of the general population, as reported by the MOH Malaysia.^12^ In Thailand, a neighbouring country of Malaysia, a comparable percentage of acceptance has also been reported (88.3%).^16^ However, this figure is high compared with that in other countries such as India (52.0%), Vietnam (60.4%) and China (77.4%).^17-19^ The high COVID-19 vaccination rate is likely a result of effective communication about the risks of complications and demonstrates trust in the MOH, emphasising the critical role of clear information. Furthermore, the widespread availability of free COVID-19 vaccines provided by the Malaysian government may have contributed to this positive response. The increased vaccination uptake may also be linked to the evolving timeline of the study, where safety concerns are progressively addressed, and more data become available over time.

In this study, 85.2% of the pregnant women had good knowledge of COVID-19 vaccination. Approximately 81.3% answered correctly that a COVID-19 vaccine is effective in reducing the severity of the infection. Patwardhan et al. also showed that 58.7% of their respondents knew that a COVID-19 vaccine could reduce the severity of the disease.^20^ The media have played a pivotal role in disseminating vital information about COVID-19 and its impact, particularly on pregnant women. By delivering current and relevant updates, the media have effectively raised awareness about the virus and its potential risks. Moreover, the media have emphasised the importance and advantages of COVID-19 vaccination, contributing to high vaccine acceptance rates among pregnant women in the community. A notable example that had a significant impact was the coverage of a well-known Malaysian singer who tragically passed away from COVID-19 while pregnant. This real-life case served as a poignant reminder of the severity of the virus’s impact on pregnant individuals, prompting heightened awareness and a greater willingness to take preventive measures, including vaccination. This highlights the media’s power in influencing the attitudes and behaviours of pregnant women towards COVID-19 vaccination. Effective communication strategies and targeted messaging are crucial to ensure informed decision-making, underscoring the importance of accurate information dissemination and awareness campaigns.

In our study, 400 pregnant women (97.1%) received COVID-19 vaccination; 76.8% of them received COVID-19 vaccination on recommendation from a doctor or the MOH. The earlier research by Patwardhan et al. during the initial phase of the COVID-19 vaccination programme found that 67.2% of pregnant women would get vaccinated if recommended by their doctors.^20^ This emphasises the significant role of healthcare providers in improving vaccine acceptability. Additionally, Lazarus et al. demonstrated that in countries with high acceptance rates, trust in the central government played a crucial role, with 48.1% of respondents stating they would get vaccinated if recommended by their employer.^21^ Two hundred ninety-nine pregnant women (72.6%) in this study agreed to get vaccinated if their employer or a higher authority mandates vaccination on them. Of the 400 vaccinated pregnant women (71%), 284 received COVID-19 vaccination voluntarily; forty were healthcare workers.

Approximately 2.9% of the respondents in this study were not vaccinated. Of them, 91.7% refused COVID-19 vaccination because of their belief that it would harm their pregnancy and baby and the lack of information regarding the safety of COVID-19 vaccination for pregnant women. Our finding is similar to the earlier reports by Upreti and Godara as well as Nguyen et al. that mentioned the same concerns raised by pregnant women who hesitated or refused to get vaccinated.^17,18^ A study in Ethiopia among the general population also showed similar results, wherein participants expressed that they were not vaccinated because of insufficient information about the vaccine.^22^ Our study showed that 210 pregnant women (51.0%) were concerned that COVID-19 vaccination would harm their pregnancy and baby. Despite their concerns, 202 of them received COVID-19 vaccination. Approximately 69% of the pregnant women believed that the vaccine effectively prevents COVID-19. Upreti and Godara reported similar concerns, with 93.1% of their respondents expressing worries about vaccine safety during pregnancy.^17^ Kumari et al. also found that 58.5% of their participants felt that a COVID-19 vaccine might have unforeseen effects on unborn children, indicating a widespread concern that needs to be addressed through education and provision of accurate information about vaccine safety.^23^

Herein, we found no significant association between acceptance and good knowledge of COVID-19 vaccination among the pregnant women (P=0.447). Approximately 85.5% of the vaccinated pregnant women and 75% of the unvaccinated pregnant women demonstrated good knowledge of COVID-19 vaccination. Uncertainties surrounding the potential risks to the foetus contribute to hesitancy among pregnant individuals, as they may be apprehensive about the vaccine’s impact on their unborn child. Only 26.9% of our respondents had tertiary education, yet a remarkable 93.7% of them exhibited good knowledge. We found a significant association between educational level and good knowledge (P<0.001); however, our analysis did not reveal a significant association between acceptance and good knowledge of COVID-19 vaccination. This finding differs from the report by Upreti and Godara, wherein acceptance and good knowledge of COVID-19 vaccination were significantly associated with higher educational levels.^17^ Mose and Yeshaneh also found that good knowledge and good practice of pregnant women towards COVID-19 and its preventive measures were significantly associated with acceptance of COVID-19 vaccination.^24^ Our study demonstrated that higher income (P=0.034) and history of receiving a COVID-19 booster dose (P=0.036) were significantly associated with acceptance of COVID-19 vaccination. This finding aligns with that of Kalok et al., wherein higher income was a significant determinant of COVID-19 vaccine acceptance.^15^ Kalok et al. also found that employment status, employment as a healthcare worker, history of COVID-19 and contact with someone who had COVID-19 were significant determinants of COVID-19 vaccine acceptance.^15^ However, we did not find any significant association between employment status, employment as a healthcare worker and history of COVID-19 and COVID-19 vaccine acceptance. Research by Dolu et al. and Malik et al. indicated that prior vaccine recipients were more inclined to accept COVID-19 vaccination.^25-26^ However, our study found no significant link between previous non-COVID-19 vaccine history and COVID-19 vaccine acceptance.

### Limitations

The study is limited by the uneven distribution of ethnicities, with a predominant Malay population, potentially affecting the generalisability of the findings. Additionally, a substantial proportion of the respondents belonged to a lower socioeconomic status, introducing potential bias.

## Conclusion

Our study found no significant association between acceptance and knowledge of COVID-19 vaccination among the pregnant women. Two factors were identified to be significantly associated with acceptance of COVID-19 vaccination: income and history of receiving a COVID-19 booster dose. Caution is needed in generalising the results to the broader Malaysian population due to the study limitations. Further research with a more diverse sample is recommended.

## Data sharing statement

Raw data are available upon request.

**How does this paper make a difference in general practice?**

- Most pregnant women in this study had a good understanding and acceptance of COVID-19 vaccination. However, safety concerns persisted, particularly among the unvaccinated pregnant women. Notably, there was no significant association found between knowledge and acceptance of COVID-19 vaccination, highlighting the necessity for disseminating comprehensive information and addressing concerns.
- This study highlights the crucial role of healthcare providers in guiding pregnant women in making informed decisions about COVID-19 vaccination. It emphasises the need for targeted education and support to address apprehensions and promote vaccination among pregnant women, thereby contributing to good maternal and foetal health during the ongoing pandemic.
